# Efficacy of subgingival irrigation with chemical agents as adjuvants to non-surgical periodontal therapy: systematic review and meta-analysis

**DOI:** 10.1007/s00784-026-06909-5

**Published:** 2026-05-13

**Authors:** Marcela Iunes da Silveira, Clarissa Carvalho Martins Maciel, Melissa M Grant, Iain L C Chapple, Andrea Carvalho De Marco, Emanuel da Silva Rovai

**Affiliations:** 1 Institute of Science and Technology, Diagnostics and Surgery Department, São Paulo State University (Unesp), Sao Jose dos Campos, Brazil; 2 School of Dentistry, Institute of Clinical Sciences, University of Birmingham and Birmingham Dental Hospital (Birmingham Community Healthcare Trust), Birmingham, UK

**Keywords:** Periodontal disease, Chemical adjuncts, Ultrasonics, Root planning, Dental scaling, Systematic review

## Abstract

**Objective:**

This systematic review aims to assess the efficacy of chemical agents (CA) in subgingival irrigation as an adjunct to non-surgical periodontal treatment (NSPT) in the treatment of periodontitis.

**Methods:**

Search strategies were developed for MEDLINE via PubMed, Web of Science, and LILACS databases for publications up to March 2025. Risk of bias was assessed according to the RoB 2.0 tool. Random-effects meta-analyses were conducted for clinical attachment level (CAL), probing pocket depth (PPD), and bleeding on probing (BOP).

**Results:**

From 1244 studies initially screened, 16 randomized clinical trials were included for qualitative and quantitative analyses. Studies assessed the effects of the following chemical agents: Povidone-iodine (PVP-I), Essential Oils (EOs); Chlorhexidine (CHX), Ozonated water (OW) and Boric Acid (BA). Overall, the meta-analysis showed that the adjunctive use of CA as subgingival irrigation did not provide additional benefit in PPD reduction, CAL gain, and BOP compared with controls (*P* > 0.05). Most studies raised some concerns with risk of bias, and 2 showed a high risk of bias.

**Conclusion:**

Adjunctive use of chemical agents in subgingival irrigation during NSPT for periodontitis patients does not appear to provide additional benefit over NSPT alone, although evidence levels are low to very low. *Clinical relevance*: CA are frequently used as adjuncts to NSPT, despite uncertainty regarding their real clinical contribution. The findings of this study help clinicians make more evidence-based decisions and avoid unnecessary use of adjunctive subgingival irrigation strategies during periodontal treatment.

**Supplementary Information:**

The online version contains supplementary material available at 10.1007/s00784-026-06909-5.

## Introduction

Periodontitis is a multifactorial chronic inflammatory disease associated with a dysbiotic biofilm and characterized by a dysregulated immune-inflammatory response, marked by significant neutrophil infiltration and the associated production of reactive oxygen species, which drives the progressive destruction of the tooth-supporting apparatus [1, 2]. The S3-level clinical guidelines on the management of stages I-IV periodontal disease are structured in sequential steps [3, 4]. Step-1 focuses on controlling risk factors, motivating the patient, and providing instructions for mechanical supragingival biofilm control, along with supragingival debridement. Step-2 consists of subgingival instrumentation, which should be performed in all patients with periodontitis who engage with step-1 of care. Subgingival instrumentation is part of nonsurgical periodontal therapy (NSPT) and aims to remove/disrupt subgingival biofilm through manual instrumentation (curettes), ultrasonic/sonic devices, or by combining both approaches [4].

Whilst NSPT remains the gold standard for controlling inflammation and halting the progression of periodontitis, specific sites or patients may respond poorly to treatment and/or fail to achieve endpoints that define disease stability, particularly in cases of severe disease and deep periodontal pockets [5–7]. Indeed, it has been estimated that only approximately 40% of periodontal pockets reach the endpoint used to designate stability, “closed pockets” (PPD ≤ 4 mm) following non-surgical treatment [6], leaving the remaining diseased sites/teeth at a higher risk of further attachment loss and tooth loss [8].

Therefore, step 3 of the treatment focuses on those sites/teeth that do not respond adequately to steps 1 and 2 (PPD ≥ 4 mm with bleeding on probing or PPD ≥ 6 mm) [4]. Adjunctive therapies employed alongside subgingival instrumentation, such as antibiotic therapy [9], laser therapy [10], and chemical agents (CA) [11], offer the potential to improve periodontal clinical parameters.

Traditionally, CAs are used in periodontal care as rinses to aid in supragingival biofilm control [12]. The primary mechanisms include preventing bacterial adhesion, inhibiting bacterial growth and/or co-aggregation, disrupting established biofilms, and altering the pathogenicity of the biofilms [11]. In this context, several studies in the literature have suggested a potential benefit from CAs used as an irrigants (delivered via syringes or as irrigants for ultrasonic scalers) in controlling the subgingival biofilm as an adjunct to NSPT [12, 13]. Clinical trials have investigated essential oils (EO) [14], chlorhexidine (CHX) [15], and povidone iodine (PVP-I) [16], as subgingival irrigants in order to promote additional clinical and microbiological benefits in the treatment of periodontitis.

Therefore, this systematic review aimed to assess the effectiveness of CA as adjuncts to NSPT in subgingival irrigation for the treatment of periodontitis.

## Materials and methods

### Protocol and registration

The present systematic review was guided and structured according to the PRISMA (Preferred Reporting Items for Systematic Reviews and Meta-analysis) guidelines and was registered at the National Institute for Health Research PROSPERO (ID: 1011516).

### Focused question

The focused question was elaborated using the PICOS acronym (population, interventions, comparisons, outcomes, study design): “In patients with periodontitis (P), does adjunctive subgingival irrigation with CA (I) during NSPT, compared with NSPT alone (C), result in additional benefits in PPD reduction and CAL gain (O), as determined in randomized controlled trials (S)?”.

### Eligibility criteria

#### (P)Population

Patients with periodontitis without systemic conditions.

#### (I)nterventions

NSPT associated with CA in subgingival irrigation (syringe or irrigation with an ultrasonic device).

#### (C)omparisons

NPST alone or using water or saline solution as a control (control group).

#### (O)utcome

The primary outcome analyzed was PPD reduction. Secondary outcomes included CAL gain and BOP change.

(S)tudy design: Only randomized clinical trials (RCT) with at least 3 months of follow-up were considered eligible for inclusion.

*Exclusion criteria*:

Studies that included pregnant women, studies with patients who had received systemic antibiotics or corticosteroids within three months prior to or during the study, studies that used gel as a vehicle for the chemical agents under investigation, studies that employed full mouth disinfection protocols, and studies that prescribed mouthwash in addition to the subgingival irrigation in the test group were excluded. Studies that used extracts or phytotherapies, studies in which follow-up was less than 3 months, or studies with insufficient information about the type of therapy assessed were also excluded from the review.

There was no language restriction.

### Information source and search strategy

Search strategies were developed for MEDLINE via PubMed, Web of Science, and LILACS databases for publications up to March 2025. Medical Subject Headings (MesH) terms were used and combined with Boolean operators (AND and OR) to search the databases. The Search strategy was as follows: (((Periodontal diseases OR Periodontitis OR periodontal disease) AND (Ultrasonic OR ultrasound OR ultrasonic OR ultrasonics or periodontal debridement or periodontal therapy or periodontal treatment or nonsurgical periodontal treatment or nonsurgical periodontal therapy or nonsurgical periodontal treatment or nonsurgical periodontal therapy or scaling and root planning)) AND (coolant OR irrigation or chemical agent or chlorhexidine or CHX or essential oils or EO or CPC or cetyl pyridinium chloride or povidone iodine or PVP-I or povidone-iodine or PVP OR sodium hypochlorite or Oxygen Water or Hydrogen peroxide) AND (randomized clinical trial or clinical study or clinical trial or RCT))).

The publications found in all electronic databases were transferred to Rayyan QCRI software (Qatar Computing Research Institute, Doha, Qatar) for selection by titles and abstracts. Electronic searches were complemented by manual searches of the reference lists of the selected articles.

### Study selection

In the first phase, two reviewers (CCMM and MIS) independently screened articles by titles and abstracts. Disagreements were resolved by discussion with a third reviewer (ESR). Then, the same reviewers (CCMM and MIS) conducted a full-text reading of the papers included in the first phase to determine eligibility.

### Data collection process

Data were independently extracted by two reviewers (CCMM and MIS) (Tables 1 and 2). If necessary, the authors were contacted to clarify data or provide any missing information.

Data were extracted by searching for the following information: (1) location where the study was conducted (2) gender and age of participants (3) length of follow-up, (4) experimental groups (5) characteristics of the compounds used (6) sample size (7) outcome measures (8) results (9) adverse events (10) Patient-Reported Outcomes (PROs).

### Risk of bias

Risk of bias was evaluated in each included study according to RoB 2.0 tool [17]. Quality assessment was performed independently by two reviewers (CCMM and MIS), with disagreements between assessors resolved by a third adjudicator (ESR).

Based on the risk of bias, inconsistency, indirect evidence, and imprecision, the quality of the evidence was assessed.

### Certainty of evidence

The GRADE (Grading of Recommendations, Assessment, Development, and Evaluation) guideline tool was used to assess the strength of evidence among randomized controlled trials regarding changes in PPD, CAL, and BOP (Table S1).

### Summary measures and synthesis of results

Analyses were performed using a software package (Revman 5.0). Random-effects or fixed-effects meta-analyses for mean differences were conducted for CAL gain, PPD reduction, and BOP. Statistical heterogeneity among the studies was assessed using the Cochran Q and and i2 statistics. Subanalyses were performed for irrigation type (syringe or ultrasonic) and follow-up.

## Results

### Search results and excluded trials

**Fig. 1:**
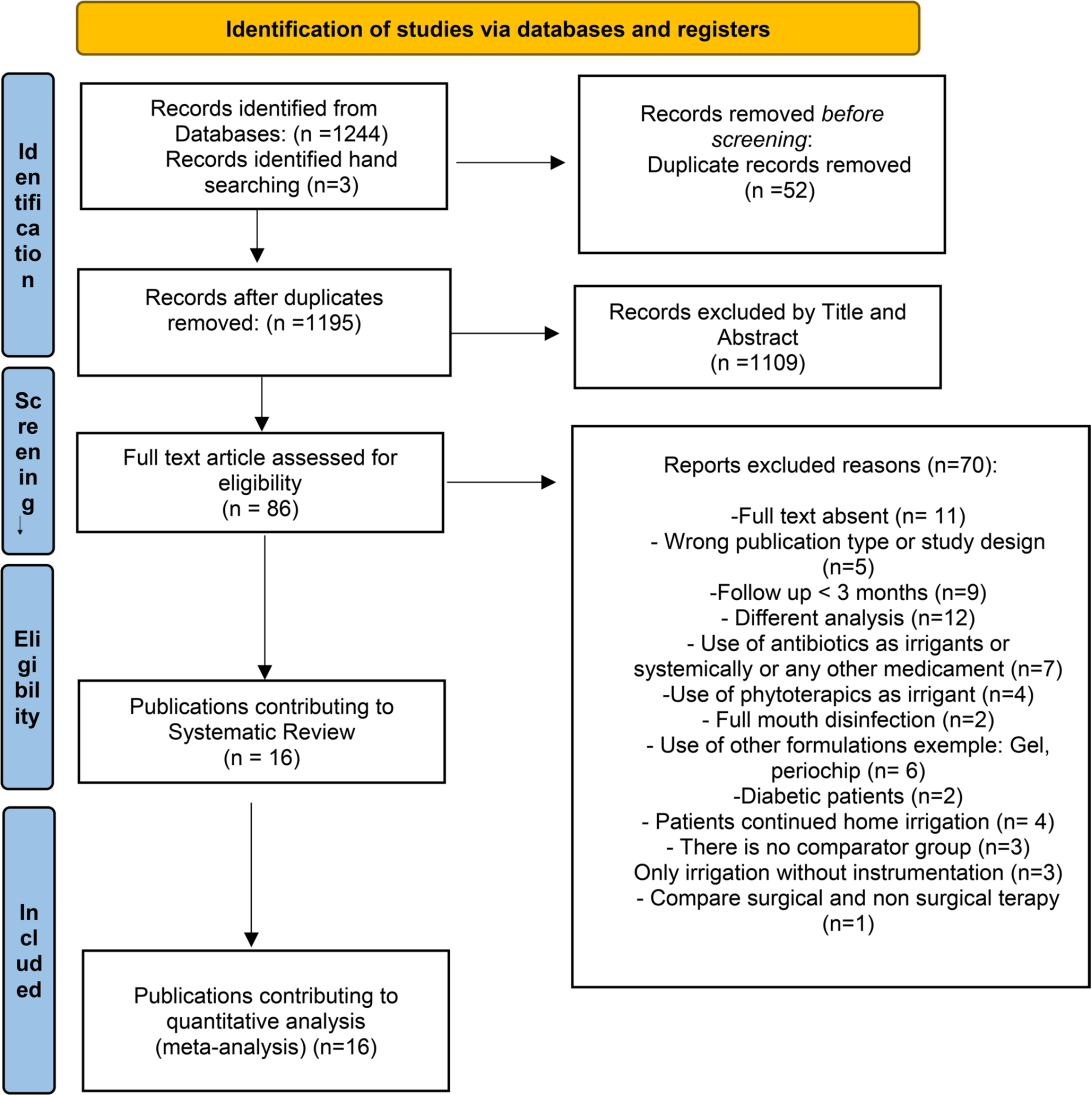
Prisma flow chart

From a total of 1244 papers identified from electronic databases and 3 from hand searching, 52 duplicate records were excluded. After screening titles and abstracts, 1109 did not meet the inclusion criteria. In the second phase of full-text reading, 70 studies were excluded. At the end, 16 publications were included in this review [14, 16, 18–31] (Fig. 1).

### Included studies

The characteristics of the included studies are documented in Table 1. From a total of 16 reports of RCTs were included in this systematic review, 13 [14, 16, 20–23, 25–31] had parallel designs and 3 used split-mouth protocols [18, 19, 24]. A total of 712 patients (336 males and 376 females) were included. The age of patients ranged from 26 to 72 years and length of follow-up ranged from 3 to 12 months (Table 1), with 7 studies following for 3 months [16, 18, 20, 22, 27, 30, 31], 6 studies for 6 months [14, 19, 21, 24, 28, 30] and 3 studies for 12 months [23, 26, 29].

**Table 1:** – Characteristics of the studies

Study	Country/ Study design	Study design	Follow-up	Sample Size	Source of Funding
Al-Saeed and Babay Nadir 2009 [18]	Saudi Arabia	Split mouth	3 months	*N* = 13 (8 Male and 5 female) Age Mean: 42.92 ± 7.55 years	Supported by College of Dentistry Research Center, Riyadh Saudi Arabia
Cosyn et al. 2013 [20]	Belgian	Parallel	3 months	*N* = 29 (17 male and 18 Female) * Age Mean: Test group: 50 ± 13 years; Control group: 44 ± 13 years	Funded by the Free University of Brussels (VUB).
Chapple et al. 1992 [19]	United Kingdom	Split mouth	6 months	*N* = 17 (8 males and 9 female) Age between: 25–65 years.	West Midlands Regional Health Authority Endowment Fund grant DAH/HMH/APP15.
do Vale et al. 2016 [28]	Brazil	Parallel	6 months	*N* = 28 (7 Male and 21 female) Age Mean: Test group: 28.54 ± 4.14 years; Control group: 28.57 ± 4.59 years	None
Feng et al. 2011 [14]	Brazil	Parallel	6 months	*N* = 64 (22 Male and 42 female) Age Mean: Test group: 54.31 ± 7.05 years Control: 54.53 ± 8.92 years	Partially supported by a grant from Johnson and Johnson Consumer and Personal Products Worldwide
Al Habashneh et al. 2015 [22]	Jordan	Parallel	3 months	*N* = 41 (13 Male and 28 Female) Age Mean: Test group 39.7 ± 13.7 years Control 39.0 ± 10.2 years	None
Krück et al. 2012 [23]	Germany	Parallel	12 months	*N* = 51 (22 Male and 29 Female) Age Mean: 51 ± 11 years	None
Leonhardt et al.2006 [24]	Sweden	Split- mouth	6 months	*N* = 20 (8 Male and 12 Female) Age Mean: 54 ± 7.7 years	None
Perrella et al. 2016 [16]	Brazil	Parallel	3 months	*N* = 29 (12 Male and 17 Female) Age Mean: test group: 43.93 ± 3.13 years control group: 44.87 ± 4.41 years	CAPES (Coordenação de Aperfeiçoamento de Pessoal de Nível Superior), (USPHS Grant DE025020).
Ribeiro et al. 2010 [25]	Brazil	Parallel	6 months	*N* = 32 (15 Male and 17 Female) Age Mean: Test group: 43.74 years Control Group: 42.96 years	None
Ribeiro et al. 2006 [21]	Brazil	Parallel	6 months	*N* = 44 (20 Male and 24 female) Age Mean: Test Group: 57/42.96 years; Control group 60/42.52 years;	Research Funding Agency from São Paulo State, Brazil (FAPESP) grant process 02/12044-8.
Rosling et al. 2001 [26]	USA	Parallel	12 months	*N* = 150 (77 Male and 73 Female) Age Mean: Test group 44.2 ± 8.8 years; Control group 44.5 ± 8.6 years	This investigation was Supported by Colgate Technology Center, N.J.USA and NIDCR(DE-12861)
Saglam et al. 2013 [27]	Turkey	Parallel	3 months	*N* = 45 (22 Male and 23 Female) Age mean – Control group:40.80 ± 7.64 years Test group 1: 43.06 ± 8.79 years Test group 2: 42.66 ± 9.30 years	This study was supported by Selcuk University Research fund Project 10,202,016 and Scientific and Technological Research Council of Turkey Project 110S502.
Vitt et al. 2019 [29]	Belarusia	Parallel	12 months	*N* = 59 (30 Male and 29 Female) Age Mean: Test group1: 46.9 ± 11.4 Test group 2 :49.4 ± 12.3 Control group: 45.4 ± 9.8	Funded by the Swedish Institute Visby Programme (Grant number 00742/2010).
Yilmaz et al. 2012 [30]	Cyprus	Parallel	3 months	*N* = 45 (28 Male and 17 Female) Age Mean: 52.8 ± 10.6 years	None
Zanatta et al. 2006 [31]	Brazil	Parallel	3 months	*N* = 45 (27 Male and 18 Female) Mean Age: Control group (29 to 62) years Test group 1: (27 to 72) years Test group 2: (27 to 59) years	None

*The study lost 6 patients and there is no data on gender of the 29 who completed follow-up

In addition, 15 studies excluded smokers, while 1 study included smokers, resulting in a total of 7 smokers among 29 patients [20] (Table 2). Table 2 depicts participants, interventions, outcomes, and results of the included studies.

**Table 2:** Participants, Interventions, outcomes and results

Study	Participants	Peridontitis definition and clinical examination	Systemic condition	Chemical agent	Type of irrigation	Interventions	Outcomes measures of interest for the review
Al-Saeed and Babay Nadir. 2009 [18]	Test group 1: N baseline = 16 N end of trial = 13 Test group 2: N baseline = 16 N end of trial = 13 Control group: N baseline = 16 N end of trial = 13	Presence of subgingival calculus and a minimum of 4 teeth in each quadrant with at least two sites with PD ≥ 4 mm and clinical attachment level ≥2 mm.	Systemically healthy patients	PVP-I 1%	Ultrasonic device	Test group: SRP + 1% PVI-I Control group: SRP + saline. Negative control: No treatment.	Test: Overall CAL change: 0.48 ± 1.49 mm Overall PPD reduction: 0.69 ± 1.13 mm Control Overall CAL change: 0.87 ± 1.50 mm Overall PPD reduction: 0.97 ± 1.25 mm
Cosyn et al. 2013 [20]	Test group: N baseline = 17 N end of trial = 12 Control group: N baseline = 18 N end of trial = 12	Chronic periodontitis, with at least one pocket per quadrant with a PPD of 6 mm or deeper showing BoP and radiographic evidence of extended bone loss (≥ one-third of the root length).	Systemically healthy patients and smokers (at least 10 cigarettes a day).	EOs	Ultrasonic device	Test group: SPR + EOs Control group: SRP + water	Test Overall PPD reduction: 0.89 ± 0.42 mm Overall CAL change: 0.48 ± 0.8 mm Overall BOP: 33 ± 18.35 (%) Control Overall PPD reduction: 1.02 ± 0.51 mm Overall CAL change: 0.48 ± 0.70 mm Overall BOP:32 ± 16 (%)
Chapple et al. 1992 [19]	Test group: N baseline = 17 N end of trial = 14 Control group: N baseline = 17 N end of trial = 14	Chronic Periodontitis in all 4 quadrants (probing depth range, 4 to 10 mm)	NI	0.2% CHX	Ultrasonic device	Test group: SPR + 0.2% Chlorhexidine Control group: SRP + water	Test Overall CAL change: 0.3 ± 0.27 mm Control Overall CAL change: 0.25 ± 0.26 mm
do Vale et al. 2016 [28]	Test group: N baseline = 17 N end of trial = 14 Control group: N baseline = 17 N end of trial = 14	Generalised aggressive periodontitis with presence of ≥ 8 teeth with PPD ≥ 5 mm with BoP and at least 2 teeth with PD ≥ 7 mm	Systemically healthy patients	PVP-I 10%	Ultrasonic device	Test group: SPR + 10% PVI-I Control group: SPR + 0.9% saline solution	Test Overall PPD reduction: 1.61 ± 0.43 mm Overall CAL change: 0.73 ± 0.88 mm Control Overall PPD reduction: 2.05 ± 0.57 mm Overall CAL change: 1.18 ± 0.85 mm
Feng et al.2011 [14]	Test group: N baseline = 32 N end of trial = 30 Control group: N baseline = 32 N end of trial = 29	Chronic periodontitis, at least one site with PPD ≥5 mm, presence of proximal attachment loss of 5 mm or more in 30% or more of teeth present.	Systemically healthy patients	EOs	Ultrasonic device, 5 min per site.	Test group: SPR + EOs Control group: SRP+ sorbitol solution 15%, ethanol 21%, sodium saccharin 0.05%, mint flavouring, green dye	Test Overall CAL change: 1.08 ± 0.65 mm Overall PDD reduction: 1.55 ± 0.60 mm Overall BOP: 41.90 ± 26.12 (%) Control Overall CAL change: 0.94 ± 0.63 mm Overall PDD reduction: 1.18 ± 0.65 mm Overall BOP: 49.24 ± 28.1 (%)
Habashneh et al. 2015 [22]	Test group: N baseline = 20 N end of trial = 20 Control group: N baseline = 21 N end of trial = 21	Chronic periodontitis, more than two interproximal sites with > 5 mm pocket depth, radiographic bone loss and > 6 mm clinical attachment loss.	Systemically healthy patients	OW	Syringe, 30–60 s.	Test group: SPR + OW Control group: SPR + irrigation of pockets with distilled water.	Test Overall CAL change: 0.4 ± 1.15 mm Overall PPD reduction: 0.8 ± 0.4 mm Overall BOP: 52 ± 23.57 (%) Control Overall CAL change: 0.6 ± 1.11 mm Overall PPD reduction: 0.4 ± 0.36 mm Overall BOP: 46 ± 26.22 (%)
Krück et al. 2012 [23]	Test group 1: N baseline = 17 N end of trial = 17 Test group 2: N baseline = 17 N end of trial = 17 Control group: N baseline = 17 N end of trial = 17	Generalized moderate chronic Periodontitis	Systemically healthy patients	0.12% CHX PVP-I 7.5%	Ultrasonic devices, 10 min per quadrant	Test group 1: SRP + 0.12% CHX Test group 2: 7.5% PVP-I Control group: saline solution	Test 1 Overall CAL change: 0.22 ± 0.64 mm Overall PPD reduction: 0.38 ± 0.41 mm Overall BOP: 18 ± 17.32 (%) Test 2 Overall CAL change: 0.36 ± 0.50 mm Overall PPD reduction: 0.44 ± 0.33 mm Overall BOP: 25 ± 17.34 (%) Control group Overall CAL change: 0.21 ± 0.70 mm Overall PPD reduction: 0.36 ± 0.38 mm Overall BOP: 16 ± 15.39 (%)
Leonhardt et al. 2006 [24]	Test group: N baseline = 20 N end of trial = 20 Control group: N baseline = 20 N end of trial = 20	One site per quadrant with the deepest periodontal pocket (greater than or equal to 6) was selected	Systemically healthy patients	PVP-I 0.5%	Ultrasonic device, 5 minutes/tooth.	Test group: SRP + 0.5% PVP-iodine Control group: SRP + saline solution	Test Overall CAL change: 0.59 ± 1.1 mm Overall BOP: 49.7 ± 23.04 (%) Control Overall CAL change: 0.69 ± 0.3 mm Overall BOP: 47 ± 24.44 (%)
Perrella et al. 2016 [16]	Test group: N baseline = 18 N end of trial = 14 Control group: N baseline = 18 N end of trial = 15	Chronic periodontitis, at least 20 teeth, who presented with a minimum of six teeth, preferably in different quadrants, that had at least one site each with probing PDD and CAL ≥ 5 mm.	Systemically healthy patients	PVP-I 10%	Syringe, for 5 min.	Test group: SRP + irrigation with 10% PVP-I, for 5 min. Control group: SRP + irrigation with saline solution	Test Overall CAL change: 0.5 ± 0.6 mm Overall PPD reduction: 0.4 ± 0.3 mm Control Overall CAL change: 0.2 ± 0.4 mm Overall PPD reduction: 0.4 ± 0.3 mm
Ribeiro et al. 2010 [21]	Test group: N baseline = 16 N end of trial = 13 Control group: N baseline = 16 N end of trial = 15	Severe chronic periodontitis, pockets with clinical attachment loss ≥ 5 mm, at least one molar with class II interproximal furcation involvement, with PPD ≥ 5 mm	Systemically healthy patients	PVP-I 10%	Ultrasonic device	Test group: SRP + 10% PVP-I for the treatment of the interproximal furcation. Control group: SRP + distilled water	Test Overall CAL change: 1.50 ± 1.09 mm Overall PPD reduction: 2.67 ± 1.21 mm Control Overall CAL change: 1.27 ± 1.02 mm Overall PPD reduction: 2.20 ± 1.10 mm
Ribeiro et al. 2006 [25]	Test group: N baseline = 24 N end of trial = 23 Control group: N baseline = 24 N end of trial = 21	Chronic periodontitis, at least one lower or upper molar with Class II furcation involvement that bled on probing in buccal or lingual surfaces with PPD ≥ 5 mm	Systemically healthy patients	PVP-I 10%	Ultrasonic device	Test group: SRP + 10% PVP-I. Control Control group: SRP + distilled water	Test Overall CAL change:6.39 ± 1.29 mm Overall PPD reduction: 2.29 ± 1.36 mm Overall BOP: 11.04 ± 10.51 (%) Control Overall CAL change:7.1 ± 1.3 mm Overall PPD reduction:2.81 ± 1.19 mm Overall BOP: 16.02 ± 10.07 (%)
Rosling et al. 2001 [26]	Test group: N baseline = 58 N end of trial = 51 Control group: N baseline = 92 N end of trial = 75	PPD of > 6 mm at > 2 teeth in each quadrant and radiographic bone loss exceeding 40% of the same teeth.	Systemically healthy patients	0,1% PVP-I	Ultrasonic device	Test group: SRP + 0.1% PVP-I Control group: SRP + water	Test Overall CAL change: 0.4 ± 0.5 mm Overall PPD reduction: 2.7 ± 0.5 mm Overall BOP: 47 ± 27 (%) Control Overall CAL change: 0.1 ± 0.5 mm Overall PPD:2.9 ± 0.6 mm Overall BOP: 41 ± 29.71 (%)
Saglam et al. 2013 [27]	Test group 1: N baseline = 15 N end of trial = 5 Test group 2: N baseline = 15 N end of trial = 15 Control group: N baseline = 15 N end of trial = 15	Chronic periodontitis, at least 20 teeth, with ≥ 8 sites with PDD ≥ 5 mm	Systemically healthy patients	0.2% CHX and 0.75% BA	Syringe,1 min per site.	Test group 1: SRP + CHX irrigation, 10 mL solution per site for 1 min Test group 2: SRP + BA irrigation 0.75%, 10 mL solution per site for 1 min Control group: SRP + saline irrigation	Test 1: Overall CAL change: 0.80 ± 0.33 mm Overall PPD reduction: 0.78 ± 0.38 mm Test 2: Overall CAL change: 0.90 ± 0.17 mm Overall PPD reduction: 0.90 ± 0.17 mm Control Overall CAL change: 0.88 ± 0.34 mm Overall PPD reduction: 0.88 ± 0.33 mm
Vitt et al. 2019 [29]	Test group 2: N baseline = 21 N end of trial = 19 Control group: N baseline = 19 N end of trial = 18	Severe chronic periodontitis, 3 teeth with periodontal pockets with a minimum PPD of 6 mm, and radiographic evidence of extensive bone loss (one-third of root length).	Systemically healthy patients	0.2% CHX	Ultrasonic device	Test group: SRP + 0.2% CHX Control group: SRP + distilled water	Test Overall PPD reduction: 1.3 ± 1.8 mm Overall BOP: 24 ± 22.11 (%) Control Overall PPD reduction: 1.3 ± 1.8 mm Overall BOP: 27 ± 17.34 (%)
Yilmaz et al. 2012 [30]	Test group 1: N baseline = 15 N end of trial = 15 Test group 2: N baseline = 15 N end of trial = 15 Control group: N baseline = 15 N end of trial = 15	Moderate to severe chronic periodontitis by the presence of periodontal pockets (≥ 5 mm) with BoP and at least one molar with Class II furcation involvement with ≥ 5 mm probing depth.	Systemically healthy patients	0,2% CHX and EOs	Ultrasonic device	Test group 1: SRP + 0.2% CHX Test group 2: SRP + EOs Control group: SRP + distilled water	Test 1 Overall CAL change: 1.8 ± 2.08 mm Overall PPD reduction: 3.34 ± 1.50 mm Overall BOP: 46 ± 10.9 (%) Test 2 Overall CAL change: 1.9 ± 1.24 mm Overall PPD reduction: 3.2 ± 1.5 mm Overall BOP: 54 ± 11.4 (%) Control Overall CAL change: 1.8 ± 1.31 mm Overall PPD reduction: 3.26 ± 1.32 mm Overall BOP: 46 ± 12.81 (%)
Zanatta et al. 2006 [31]	Test group 1: N baseline = 15 N end of trial = 15 Test group 2: N baseline = 15 N end of trial = 12 Control group: N baseline = 15 N end of trial = 13	Moderate to Severe chronic periodontitis. Exhibited at least eight teeth with periodontal lesions characterized by a PDD ≥ 5 mm and bleeding on pocket probing.	Systemically healthy patients	PVP-I 0.5% and NaCl	Ultrasonic device	Test group 1: SRP + 0.5% PVP-I Control: SRP + 0.9% NaCl.	Test 1 Overall PPD reduction: 2.53 ± 0.50 mm Overall CAL change: 1.94 ± 0.70 mm Control Overall PPD: 2.58 ± 0.60 mm Overall CAL: 1.99 ± 0.92 mm

Legend: PPD Probing pocket depth, CAL Clinical Attachment Level, BoP Bleeding on probing, SRP Scaling and root planing, PVI-I Iodo Povidine, CHX Chlorhexidine, OW Ozonated water, EOs Essential Oils, NaCl sodium chloride, BA Boric acid, mm milimeters, N number

Regarding the irrigation methods, 13 of the included studies used ultrasonic scalers with a CA as irrigator (14,18–21,23–26,28–31) while the other 3 studies used syringe-delivered irrigation [16, 22, 27]. Additionally, two studies used ultrasonics supplemented by manual instrumentation, but the CA in both studies was used only in a syringe [22, 29].

The types of CA were: PVP-I in concentrations of 10% [16, 25, 28] (*n* = 3), 1% (1) (*n* = 1), 0.5% [24, 31] (*n* = 2), 0.1% (23) (*n* = 1) and 1 study did not report the concentration used (6); essential oils (EOs) [14, 20, 30](*n* = 3); these were identified in three different formulations, the first being containing 0.064% thymol, 0.092% eucalyptol, 0.06% methyl salicylate, 0.042% menthol and 21.6% ethanol) [30] (*n* = 1), followed by menthol (0.042%), thymol (0.064%), methyl salicylate (0.060%) and eucalyptol (0.092%) [20] (*n* = 1), and also 0.064% thymol, 0.092% eucalyptol, 0.06% methyl salicylate, 0.042% menthol, and 21.6% ethanol) [14] (*n* = 1); CHX in concentrations of 0.2% (4,24,35,36) (*n* = 4) and CHX in concentration of 0.12% (*n* = 1) [16]; BA at a concentration of 0.75% [27] (*n* = 1); and OW [22] (*n* = 1).

### Risk of bias

**Fig. 2:**
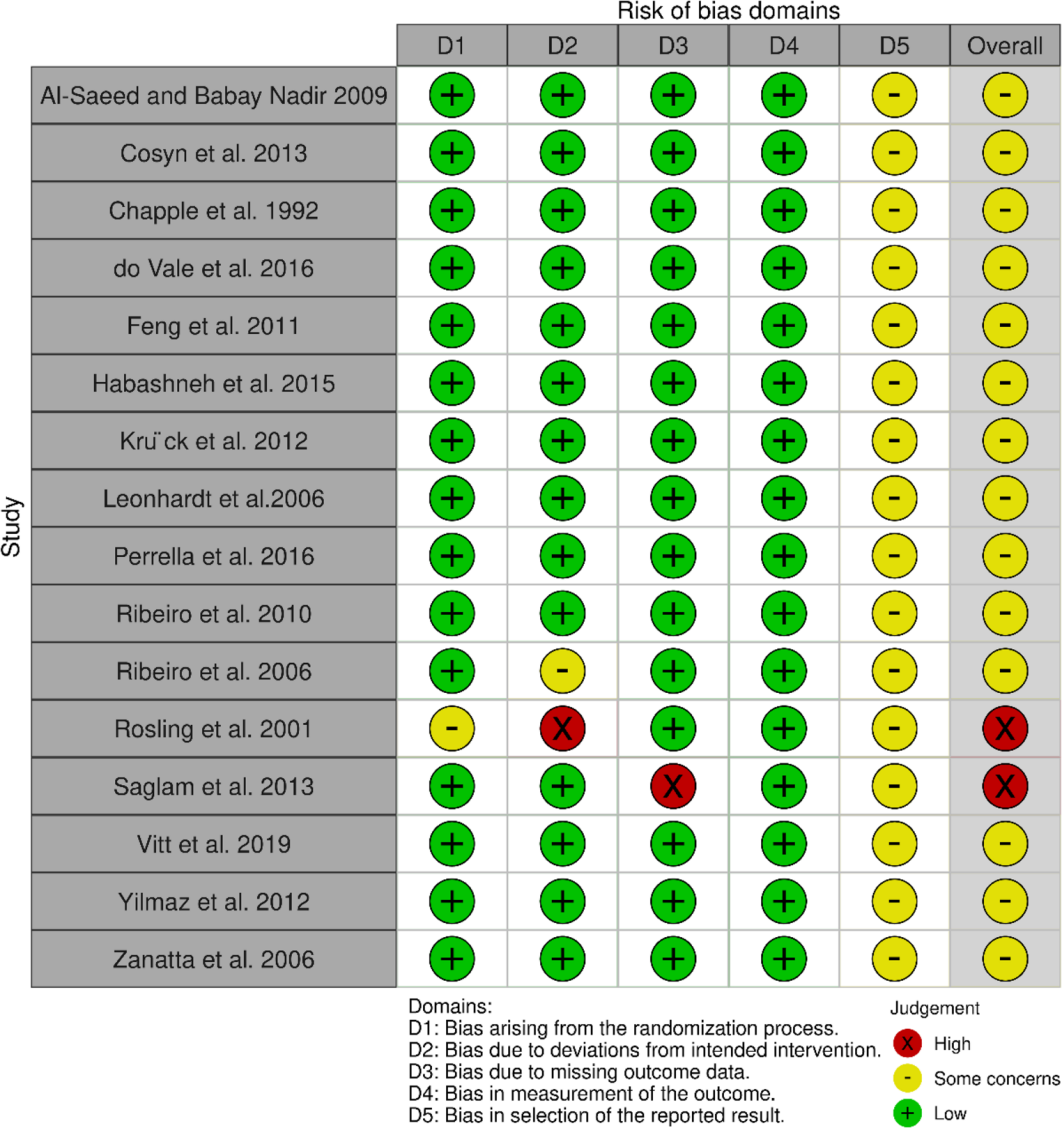
Risk of bias using the RoB2 tool for randomized controlled trial studies

From 16 RCT, 14 studies were considered to have some concerns over risk of bias [14, 16], 18– [21–25, 28, 29, 31] mainly due to issues in Domain 5 (deviations from the reported results), as none of these studies reported prior registration. The remaining two studies were rated as high risk of bias, Rosling et al., 2001 and Saglam et al., 2013. Rosling et al., 2001 was at high risk in Domain 2 (deviations from the intended interventions) with some concerns in Domain 1 (randomization process) and Domain 5 (deviations from the reported results) [26], while Saglam et al., 2013. a high risk of bias in Domain 3 (missing outcome data) and Domain 5 (deviations from the reported results) [27] (Fig. 2).

### Meta-analysis

Meta-analysis were conducted with data from the 16 RCTs [14, 16, 18–31]. Sub-analyses were performed for CA type, irrigation method, and follow-up time.

In addition, separate analyses were conducted for three studies that only included teeth with furcation involvement [21, 25, 30].

**Fig. 3:**
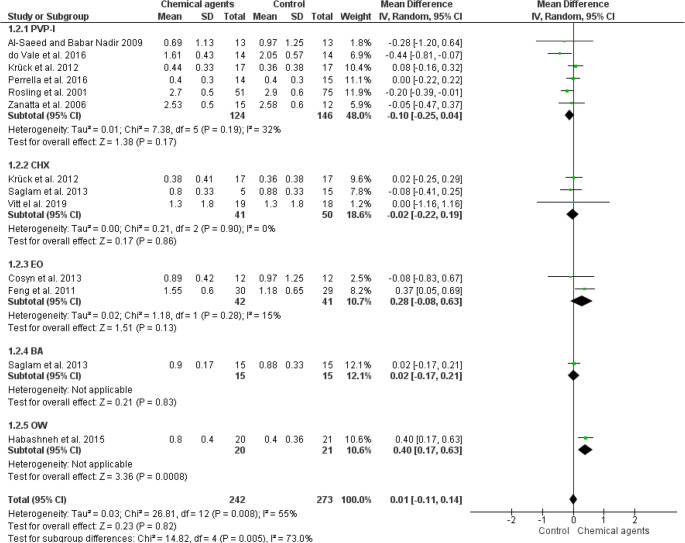
Forest plot of fixed-effects meta-analyses evaluating PPD change in patients with periodontitis treated with NSPT combined with the use of different types of CA as irrigators and a control. Legend: CHX (clorexidine); CI (confidence interval); EO (Essential oils); PVP-I (povidone iodine); BA (boric acid); OW (ozonated water)

**Fig. 4:**
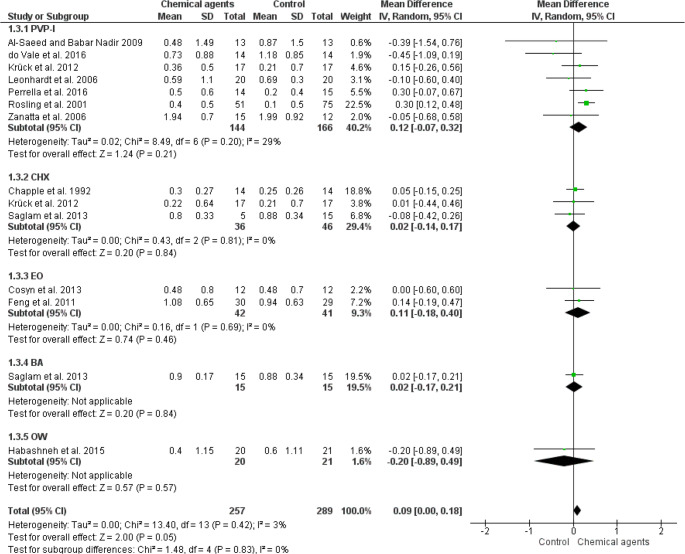
Forest plot of fixed-effects meta-analyses evaluating CAL in patients with periodontitis treated with NSPT combined with the use of different types of CA as irrigators and a control. Legend: CHX (clorexidine); CI (confidence interval); EO (Essential oils); PVP-I (povidone iodine); BA (boric acid); OW (ozonated water)

**Fig. 5:**
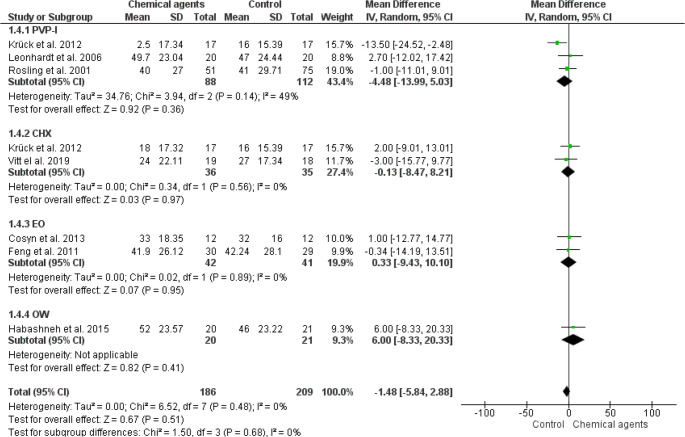
Forest plot of fixed-effects meta-analyses evaluating the change in the number of sites with BOP in patients with periodontitis treated with NSPT combined with the use of different types of CA as irrigators and a control. Legend: CHX (clorexidine); CI (confidence interval); EO (Essential oils); PVP-I (povidone iodine); BA (boric acid); OW (ozonated water)

Overall meta-analysis of the 13 studies [14, 16, 18, 19, 22–24, 26–29, 31] indicated no significant differences in PPD change (0.01; IC from 95%: -0.11, 0.14; *p* = 0,82; I² 73%) (low quality of evidence: Fig. 3, table S1), CAL gain (0.09; IC from 95%: 0.00, 0.18; *p* = 0.05; I² 0%) (low quality of evidence: Fig. 4, table S1) and BOP reduction (-1.48; IC from 95%: -5.84, 12.88; *p* = 0.51; I² 0%) (low quality of evidence: Fig. 5, table S1) in the use of CA as an adjunct to NSPT when compared to control (water or saline solution) (Figs. 3, 4 and 5). In addition, PVP-I, CHX, EO, and BA did not provide additional benefits to NSPT compared with control irrigants in any periodontal clinical parameter. However, the subanalysis with only one study [22] indicated a slight but significant reduction in PPD associated with the use of OW compared to the control.

Regarding the follow-up periods, no significant differences were found in PPD reduction or BOP changes at any follow-up period (Figures S1 and S3). For CAL gain, the subgroup analysis restricted to studies that did not evaluate furcation involvement and at 12 months of follow-up revealed a small but significant improvement in the test group (0.25; 95% CI: 0.09–0.40; *p* = 0.002; I² = 0%), which was not observed at 3 or 6 months (Figure S2). Additionally, subgroup analyses of irrigation methods (syringe or ultrasonic irrigation) revealed no significant differences in periodontal clinical outcomes, including PPD reduction (Figure S4), CAL gain (Figure S5), and BOP change (Figure S6), regardless of the method used.

In the meta-analyses of three studies evaluating teeth with furcation involvement [21, 25, 30], the use of different CA showed no significant differences between groups for PPD reduction (0.02; 95% CI: − 0.44 to 0.48; *p* = 0.92; I² = 0%) (Figure S7), CAL change (–0.08; 95% CI: − 0.53 to 0.38; *p* = 0.74; I² = 0%) (Figure S8), or BOP reduction (0.56; 95% CI: − 6.98 to 8.11; *p* = 0.88; I² = 64.5%) (Figure S9).

Further, the funnel plot analysis did not indicate substantial publication bias in the overall meta-analysis from PPD reduction (Figure S10), CAL gain (Figure S11) and BOP change (Figure S12).

### Additional clinical outcomes

In addition to mean full-mouth PPD and CAL changes, some studies also reported clinically relevant outcomes related to the presence of residual periodontal pockets and their proportions following NSPT [18, 21, 22, 24–26, 29]. Overall, a reduction in the proportion of sites with greater probing depths after treatment was observed across all evaluated groups, with no significant differences between the tested CA.

Al-Saeed et al. observed significant reductions in the percentage of sites with PD > 3 mm and < 6 mm in all treated groups, including PVP-I (25.57 ± 43.6) and saline solution (21.74 ± 41.30), with no statistically significant differences between them [18]. Similar findings were reported by Habashneh et al., who demonstrated a significant reduction in the percentage of sites with PD ≥ 4 mm after 3 months in both the OW group (4.06%) and the control group (4.06%) [22]. Likewise, Vitt et al. reported a significant reduction in the number of PD > 4 mm after 12 months, with no differences between the experimental groups (CHX- test group − 1.4 ± 1.7 and water- control group − 1.3 ± 1.5) [29].

Regarding the proportion of sites that achieved PD compatible with pocket closure after treatment, Leonhardt et al. reported that after 6 months, 47.4% of sites treated with ultrasonic scaling combined with PVP-I irrigation and 57.9% of sites treated with ultrasonic scaling combined with saline irrigation presented PD < 4 mm [24].

The presence of deep residual periodontal pockets associated with furcation defects was also investigated. Ribeiro et al. conducted two studies evaluating teeth with class II or III furcation involvement [21, 25]. No statistically significant differences were observed between ultrasonic instrumentation combined with 10% PVP-I and distilled water regarding the percentage of sites with PD ≥ 5 mm (70.00% in the control group and 36.84% in the test group) [21] or regarding the need for retreatment after 6 months (16.13% in the control group and 11.14% in the test group in the 2006 study [25]; 33.33% in the control group and 15.79% in the test group [21].

Finally, when analyzing the presence of deep residual pockets, Rosling et al. demonstrated an overall reduction in the proportion of periodontal pockets over 12 months, with a high percentage of sites presenting PD < 3 mm (79% ± 16% in the test group and 74% ± 19% in the control group) and a low frequency of sites with PD > 7 mm in both groups (2% ± 4% in the test group and 5% ± 7% in the control group) [26]. Similarly, Vitt et al. also reported no differences between 0.2% CHX and water regarding the reduction in the number of deep pockets (≥ 6 mm) after 12 months (− 1.7 ± 1.9 in the test group and − 1.7 ± 1.7 in the control group), although a reduction compared with baseline was observed in both groups [29].

### Adverse effects and PROs

Regarding the presence of adverse effects, among the 16 studies included in this review, only two reported such events [14, 20]. Cosyn et al. reported related pain, tooth hypersensitivity and recurrent oral ulcerations in both the control group (water) and the test group (EO) [20]. In the study by Feng, dental sensitivity was reported one week after ultrasonic instrumentation sessions in both the test group (EO) and the control group [14]. Of the remaining 14 studies, four reported no adverse events [16, 23, 27, 29] while the others did not mention anything regarding adverse effects [18, 19, 21, 22, 24–26, 28, 30, 31].

Only one of the studies included in this systematic review assessed PROs [19]. In this study, patient discomfort was assessed for all participants using a 10-cm visual analogue scale (VAS) at the end of the treatment modalities. The results showed no difference between the groups (CHX or water) [19].

## Discussion

This systematic review identified 16 RCTs that evaluated the clinical efficacy of chemical adjuncts as a subgingival irrigants during NSPT, compared with a control irrigant of water or saline. The overall results demonstrated that the adjunctive use of CA as a subgingival irrigant in NSPT did not provide additional benefit over the control irrigant in periodontal clinical parameters such as PPD reduction, CAL gain, or BOP change.

The chemical adjuncts employed as irrigants in the studies analysed within this systematic review were PVP-I, CHX, EO, OW, and BA used adjunctively to NSPT. A subgroup analysis by CA revealed no additional benefit in periodontal clinical parameters associated with PVP-I, CHX, EO, or BA compared to the control irrigants. The 2016 systematic review by Van der Sluijs et al. largely corroborates our findings; however, they reported a slight but significant CAL gain associated with subgingival irrigation using PVP-I, a finding that was not confirmed in the present review [32]. Although published nearly a decade ago, Van der Sluijs’ review remains the most recent on this topic, highlighting the need for an updated analysis. The inclusion of 8 additional RCTs, together with the stricter eligibility criteria applied in the present review, may explain the discrepancies between our findings and those reported previously by the above-mentioned study [16, 21–23, 25, 29, 30]. Specifically, studies with follow-up periods shorter than 3 months were excluded, as such durations are insufficient to assess periodontal outcomes following an appropriate period of healing [33–35]. Studies in which mouthwashes were used in addition to subgingival irrigation with CAs for a period after NSPT [36, 37] were excluded, as this adjunctive use could confound the attribution of outcomes to subgingival irrigation alone. Finally, the study by Forabosco (2006) was excluded because the data were not accessible for analysis [38]. Another distinguishing feature of the present review compared with the previously mentioned one is that subgroup analyses were performed according to the type of irrigant, irrigation method (syringe vs. ultrasonic), follow-up duration, and the presence of furcation involvement, allowing a more detailed evaluation of potential factors influencing treatment outcomes. Additionally, data on adverse effects, the presence of any PRO analyses, and the prevalence and proportion of residual periodontal pockets following treatment were extracted from the included studies.

The use of CA as mouthwashes in periodontal therapy is well established for controlling supragingival plaque and inflammation [12]. However, the distinct characteristics of supragingival and subgingival microbiota composition, maturation, and maintenance have been well-documented in the literature [39]. This highlights that the effectiveness of an agent in the supragingival environment does not necessarily translate to the subgingival environment, where conditions are more complex due to the intimate interactions with periodontal tissues, gingival crevicular fluid flow, which washes out the irrigant, and the host immune response. Therefore, slow-release systems, such as Periochip and Arestin, have been investigated due to their high substantivity [40]. However, according to the Treatment of Stage I–III Periodontitis — The EFP S3 Level Clinical Practice Guideline, there are no significant long-term effects, in addition to insufficient data on CAL and BOP, and no data on pocket closure with the use of those agents [3].

Based on a single study, a subanalysis suggested a potential small benefit in PPD reduction associated with subgingival irrigation using OW [22]. However, these findings should be interpreted with caution due to the limited number of included studies and the observed PPD reduction of 0.40 mm, which may not be clinically significant. It is also noteworthy that, whilst the present systematic review’s individual analysis suggested a difference, the original RCT’s statistical analysis did not find a significant effect. This discrepancy may be explained by differences in statistical approaches: effect size estimation versus direct group-mean comparisons in the abovementioned RCT.

An additional subanalysis was conducted for the irrigation method (syringe or ultrasonic device); however, the meta-analysis showed no additional benefit of CA, regardless of the irrigation method used. It is worth noting that syringe irrigation may allow for greater standardization of the agent volume and more precise delivery, particularly in deep pockets, compared to ultrasonic irrigation. Regarding ultrasonic devices, although irrigation time can be standardized, the direction and distribution of the irrigant may vary depending on the type and brand of the tips employed or the device used.

Duration of follow-up in periodontal treatment studies is an important consideration, as short-term results are not always sustained over time [5]. Our analysis across different follow-up periods showed a small but statistically significant difference in CAL gain at 12 months. Nonetheless, this finding should be interpreted with caution, given that only two studies included a one-year follow-up [23, 26], and one of them had an unequal number of participants between the control and the CA-treated groups [26]. No differences were observed for the other periodontal clinical parameters across the 3, 6, and 12-month follow-up periods. Most studies included had a short-term follow-up, in reinforcing the need for more studies with long follow-up periods.

Analyses focused on outcomes directly reported by patients remain scarce in the literature, with only a single study reporting a PRO using a visual analogue scale (VAS) [19], which indicated no additional discomfort associated with the use of CA during NSPT. Notably, no study employed validated patient-reported outcome measures (PROMs), such as oral health-related quality of life instruments, limiting the ability to comprehensively assess the impact of these interventions from the patient’s perspective. Given that VAS represents a single-item measure and may not capture the multidimensional nature of patient experience, the current evidence provides only a partial understanding of patient-centred outcomes. This highlights a significant gap in the literature and underscores the need for future studies incorporating validated PROMs to better inform clinical decision-making.

The risk of bias in the studies included in this review was considered moderate, as none of the studies reported protocol registration on RCT platforms, a factor that may increase the risk of selective reporting and compromise methodological transparency. However, it should be acknowledged that some of the included studies were conducted and published before the widespread adoption of prospective trial registration and the consolidation of international recommendations encouraging protocol registration, such as those proposed by PRISMA and clinical trial registries. Furthermore, the overall risk of bias in some of the included studies was high, which may have significantly influenced the results presented in this review.

High heterogeneity (≥ 70%) was observed for some analyses (specially PPD reduction). This can be attributed to variations in the number of studies per CA, differences in concentrations, and methodological discrepancies, such as including data from all sites versus only deep pockets. Some studies presented their results stratified by PD (moderate or severe sites), whereas others did not, reporting only the mean difference in full-mouth PD data. This approach may mask clinical outcomes, as mean values of clinical parameters are diluted by healthy sites where no clinically significant post-treatment changes are observed. Additionally, a lack of standardization in the irrigation method (volume and duration) was noted. The heterogeneity of BOP in the subanalysis of teeth with furcation involvement can also be explained by the limited number of studies addressing this outcome measure.

Notably, given concerns regarding microbial resistance and tolerance, the use of antiseptics, particularly CHX, should be approached with caution. Repeated exposure may promote microbial adaptation and reduced susceptibility, particularly within biofilms, where limited penetration can lead to sublethal exposure [41]. Although the clinical relevance of these effects remains uncertain, such concerns, together with the lack of demonstrated additional clinical benefit, do not support the routine use of these agents as adjunctive subgingival irrigants to NSPT and should be carefully considered in light of current antimicrobial stewardship principles [42]. These findings further highlight the need for well-designed clinical studies to better establish their role in periodontal therapy.

The results of this review should be interpreted with caution due to several limitations. The most reported outcomes (PPD reduction and CAL gain) were expressed as full-mouth means, which may not adequately reflect treatment success at the patient or site level. The lack of standardization in the definition and reporting of residual pockets also precluded meta-analysis of these outcomes, representing an important limitation of the available evidence. Future studies should therefore assess clinically relevant endpoints at both patient and site levels (e.g., pocket closure, PPD < 4 mm), as recommended by international guidelines. Standardization of irrigant volume and application time, as well as longer follow-up periods, is also needed. Furthermore, studies evaluating PROMs are essential to better capture the patient-centered impact of these interventions.

There is still room for future exploration of the use of CA as subgingival irrigants adjunctive to NSPT, particularly through the development of novel agents, formulations, delivery methods, or protocols aimed at enhancing clinical benefits while reducing the need for periodontal surgery and the use of antibiotics in periodontal therapy.

## Conclusion

### *Implications for clinical practice*

In this systematic review and meta-analysis, low to very low evidence suggests that the adjunctive use of CA as subgingival irrigant in patients with periodontitis did not demonstrate additional benefits compared to NSPT alone regarding CAL gain, PPD reduction, and BOP. 

### *Implications for research*

This is an updated evidence on the use of CA as adjuntos to non surgical periodontal therapy. Further research is needed to explore the use of CA as an adjunctive subgingival irrigants in NSPT, focusing on novel agents, formulations, and delivery strategies to enhance clinical outcomes and reduce the need for periodontal surgery and antibiotics.

## Supplementary Information

Below is the link to the electronic supplementary material.

Supplementary Material 1 (DOCX 424 KB)

## Data Availability

No datasets were generated or analysed during the current study.
