# Supplementary material for: Efficacy of subgingival irrigation with chemical agents as adjuvants to non-surgical periodontal therapy: systematic review and meta-analysis
Source: Clin Oral Investig. 2026 May 13;30(6):227. doi: 10.1007/s00784-026-06909-5 (PMC13171656; doi:10.1007/s00784-026-06909-5)

**SUPPLEMENTARY MATHERIAL**

**Table S1** GRADE Summary table of results for the impact on periodontitis treatment of the use of different chemical agents in subgingival irrigation as adjuvants to nonsurgical periodontal therapy.

| **Certainty assessment** | | | | | | | **Effect** | **Quality of Evidence** | **Importance** |
| --- | --- | --- | --- | --- | --- | --- | --- | --- | --- |
| **№ of studies** | **Study design** | **Risk of bias** | **Inconsistency** | **Indirectness** | **Imprecision** | **Other considerations** | **Mean Difference (95% CI)** |  |  |
| **PPD change** | | | | | | |  |  |  |
| 11 | Randomized trial | Serious ^a^ | Serious ^b^ | Not serious | Serious^c^ |  | mean **0.01 mm** (-0.11 lower to 0.14 higher) | Low  ^a.b.c^ |  |
| **CAL change** | | | | | | |  | | |
| 12 | Randomized trial | Serious^a^ | Not serious | Not serious | Serious^c^ |  | mean **0.09 mm**  (0.00 lower to 0.18 higher) | Low  ^a,c^ |  |
| **BOP change** | | | | | | |  | | |
| 7 | Randomized trial | Serious^a^ | Not serious | Not serious | Very serious^d^ |  | mean **-1.48 mm**  (-5.84 lower to 2.88 higher) | Very Low  -^a,d^ |  |

**CI:** confidence interval

a. The proportion of information from studies at high risk of bias is insufficient to affect the interpretation of results. Most studies were classified as presenting some overall concerns about the risk of bias.

b. Substantial heterogeneity (I^2^ 77.2%) was found.

c. The CI cross the non-difference line.

d. The CI cross the non-difference line and the number of participants is less than the minimum information size for quantitative data.

**Fig. S1** Forest plot of fixed-effects meta-analyses evaluating PPD change in patients with periodontitis treated with NSPT combined with the use of different types of CA as irrigators in different follow-ups.


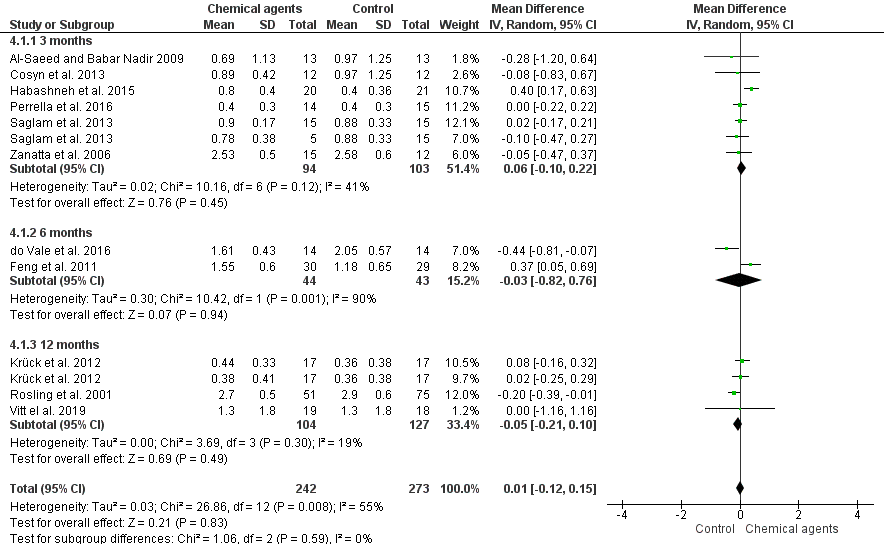


Legend: CI- confidence interval

**Fig. S2** Forest plot of fixed-effects meta-analyses evaluating CAL change in patients with periodontitis treated with NSPT combined with the use of different types of CA as irrigators in different follow-ups.


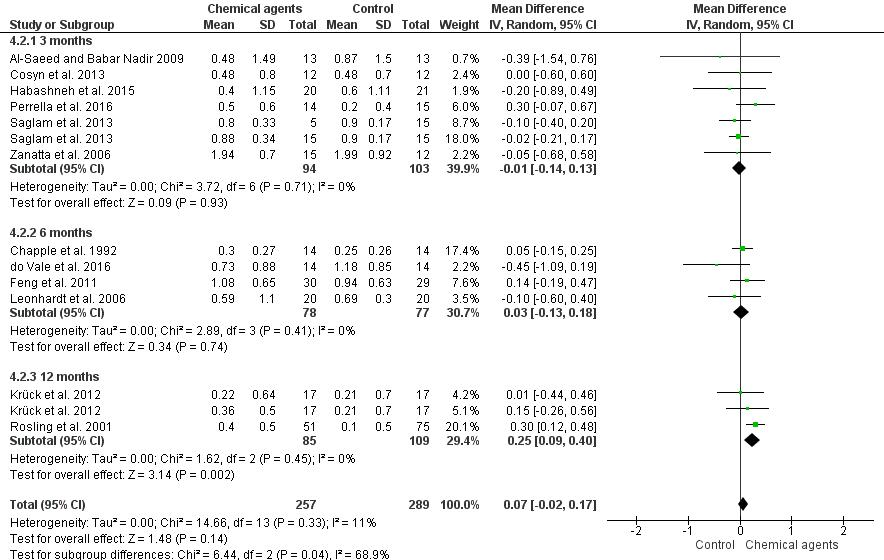


Legend: CI- confidence interval

**Fig. S3** Forest plot of fixed-effects meta-analyses evaluating BOP in patients with periodontitis treated with NSPT combined with the use of different types of CA as irrigators in different follow-ups.


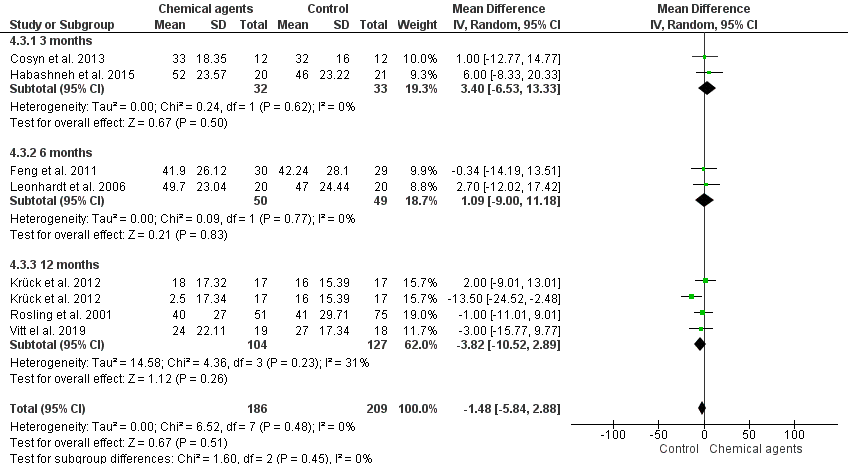


Legend: CI- confidence interval

**Fig. S4** Forest plot of fixed-effects meta-analyses evaluating PPD change in patients with periodontitis treated with NSPT using different irrigation methods (syringe or ultrasonic device).


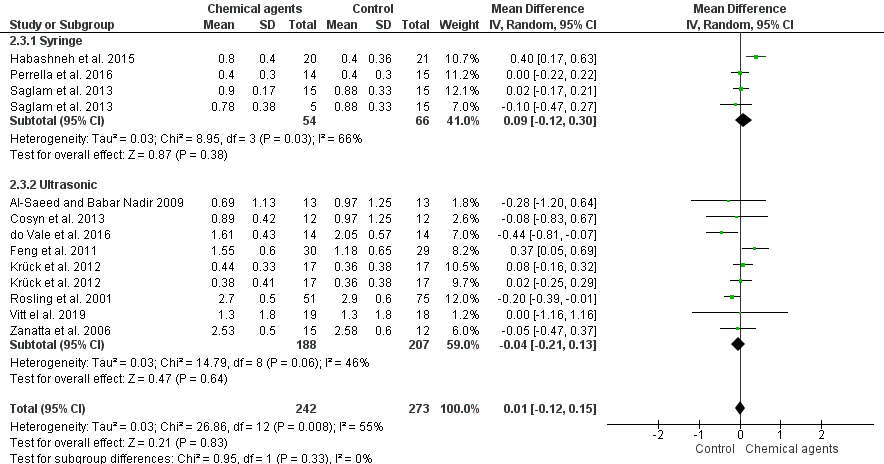


Legend: CI (confidence interval)

**Fig. S5** Forest plot of fixed-effects meta-analyses evaluating CAL gain in patients with periodontitis treated with NSPT using different irrigation methods (syringe or ultrasonic device).


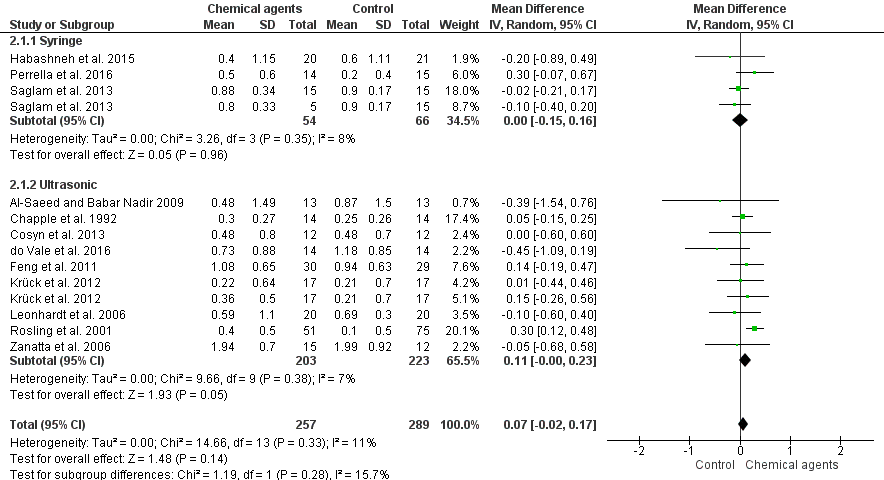


Legend: CI (confidence interval)

**Fig. S6** Forest plot of fixed-effects meta-analyses evaluating BOP in patients with periodontitis treated with NSPT using different irrigation methods (syringe or ultrasonic device).


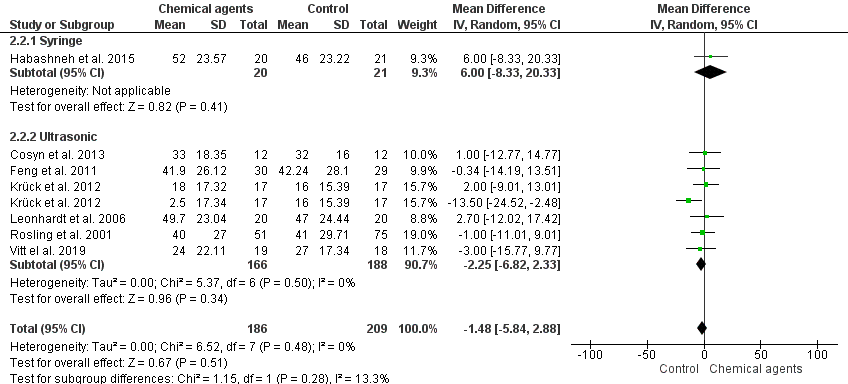


Legend: CI (confidence interval)

**Fig. S7** Forest plot of fixed-effects meta-analyses evaluating PPD change in patients with periodontitis in teeth with furcation involvement, treated with NSPT combined with the use of different types of CA as irrigators.


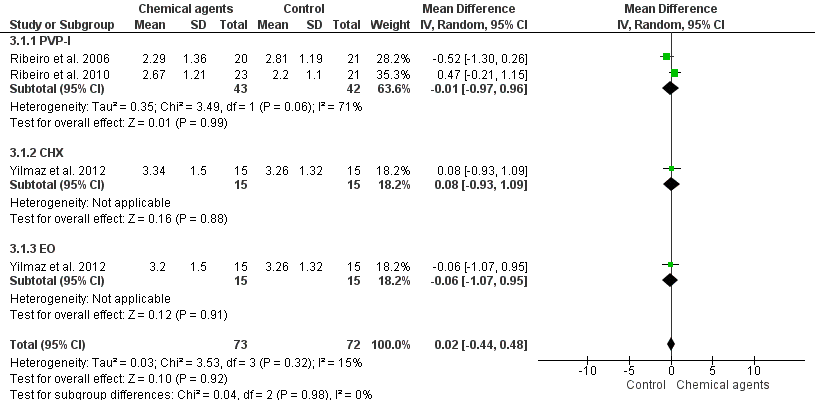


Legend: CHX (clorexidine); CI (confidence interval); EO (Essential oils); PVP-I (povidone iodine); BA (boric acid); OW (ozonated water).

**Fig. S8** Forest plot of fixed-effects meta-analyses evaluating CAL change in patients with periodontitis in teeth with furcation involvement, treated with NSPT combined with the use of different types of CA as irrigators.


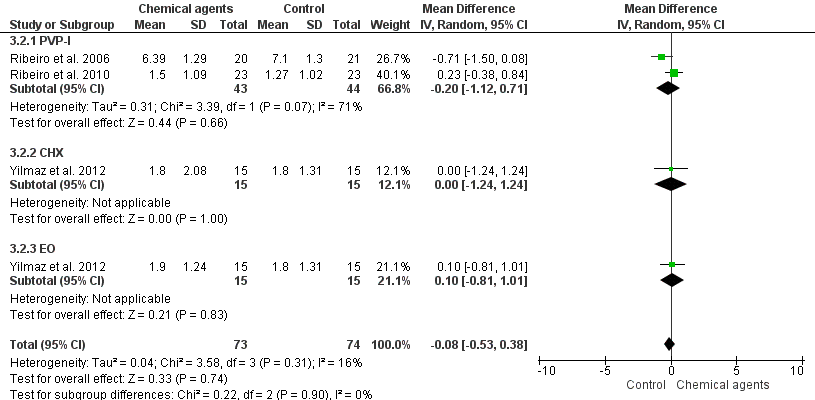


Legend: CHX (clorexidine); CI (confidence interval); EO (Essential oils); PVP-I (povidone iodine); BA (boric acid); OW (ozonated water).

**Fig. S9** Forest plot of fixed-effects meta-analyses evaluating BOP reduction in patients with periodontitis in teeth with furcation involvement, treated with NSPT combined with the use of different types of CA as irrigators.


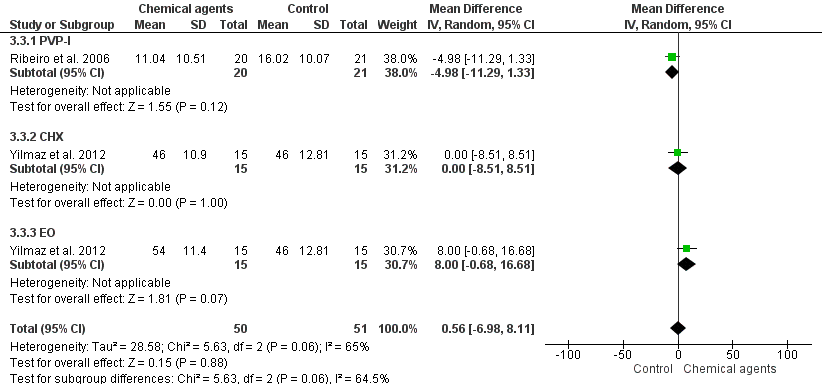


Legend: CHX (clorexidine); CI (confidence interval); EO (Essential oils); PVP-I (povidone iodine); BA (boric acid); OW (ozonated water).

**Fig. S10** Funnel plot of meta-analysis evaluating PPD reduction


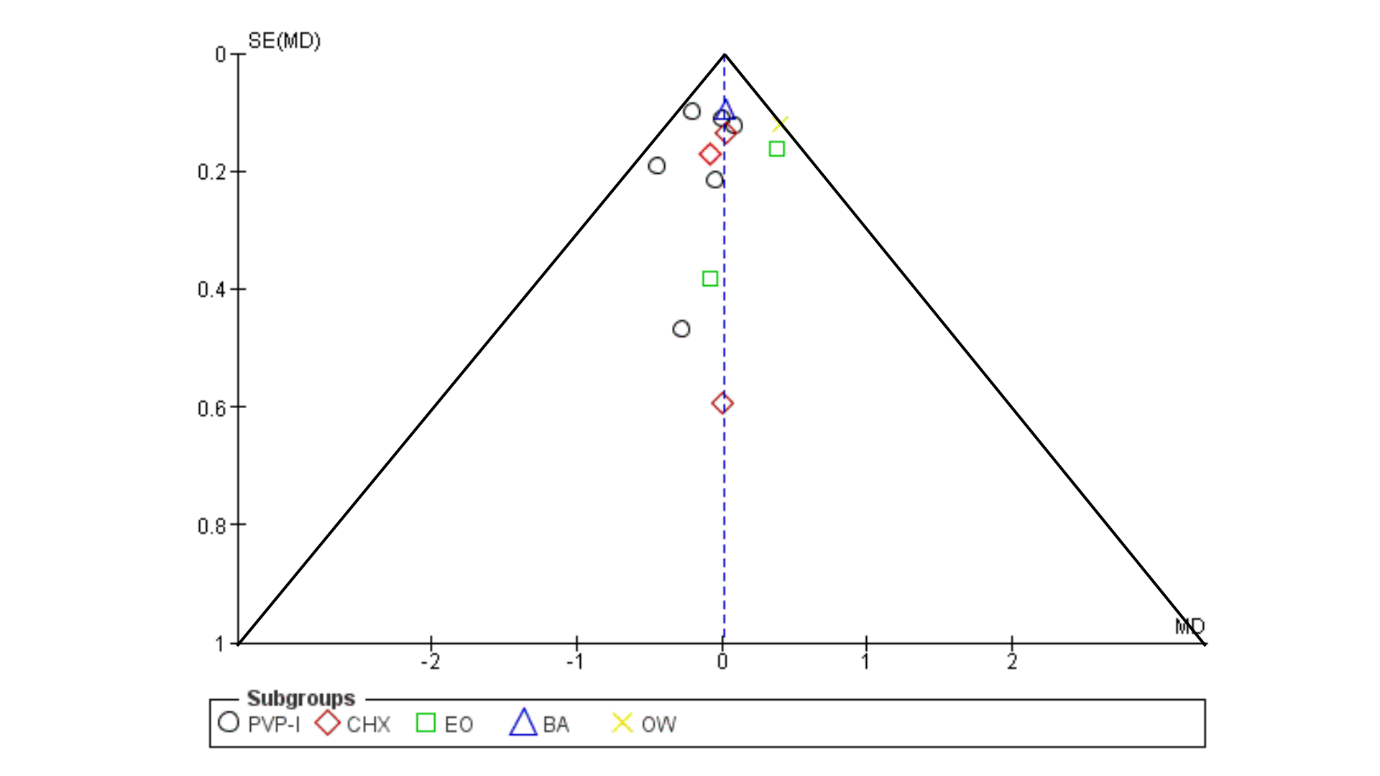


**Fig. S11** Funnel plot of meta-analysis evaluating CAL gain


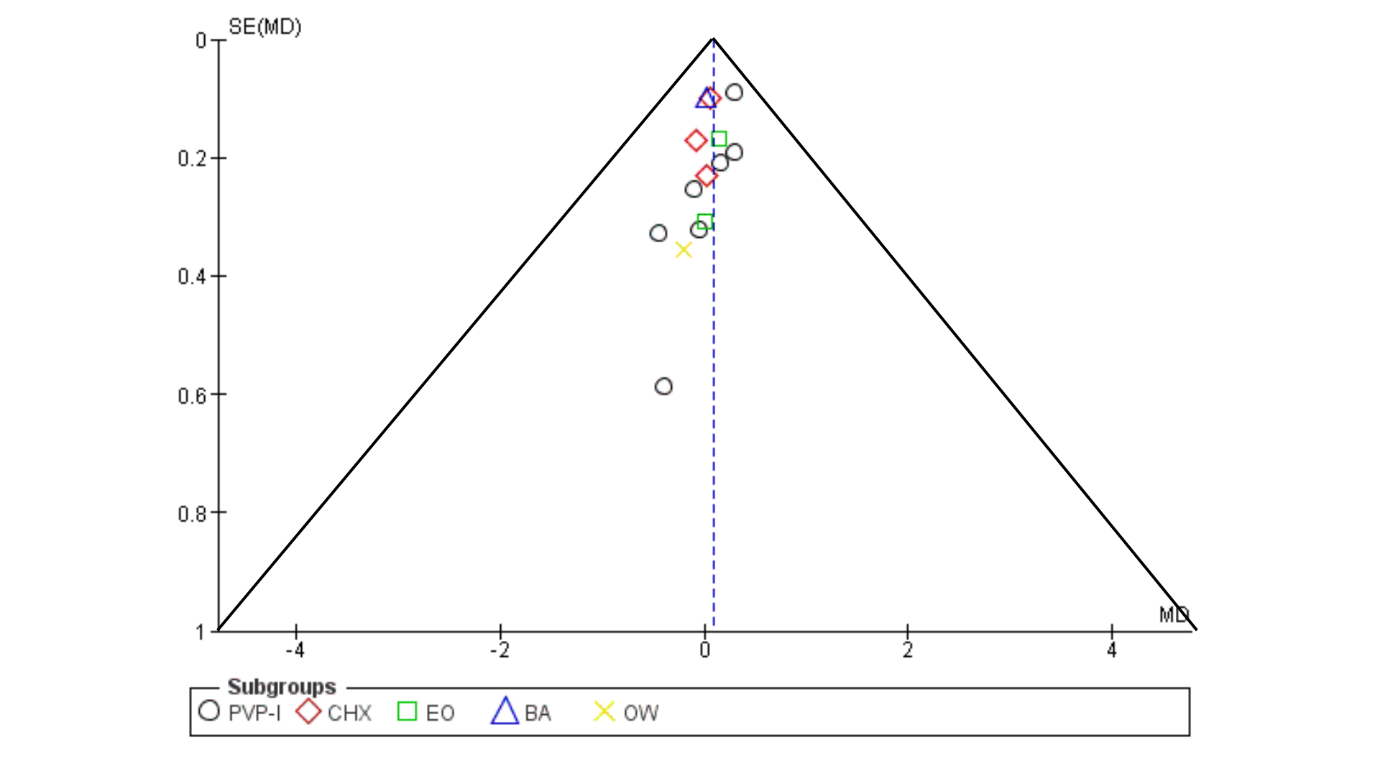


**Fig. S12** Funnel plot of meta-analysis evaluating BOP


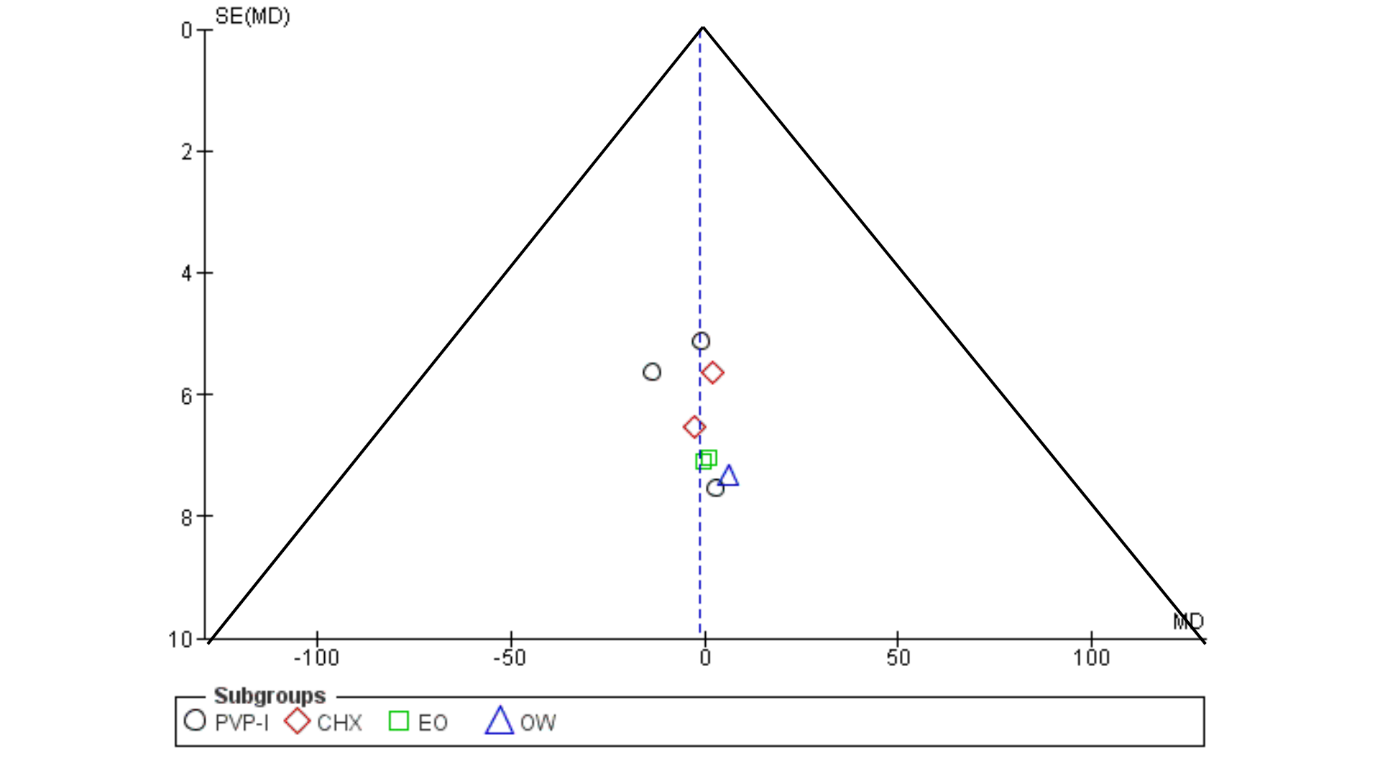

Supplement: Supplementary file 1 — Supplementary Material 1 (DOCX 424 KB) [file 784_2026_6909_MOESM1_ESM.docx]
